# Drug Reaction With Eosinophilia and Systemic Symptoms Secondary to Trimethoprim/Sulfamethoxazole: A Case Report

**DOI:** 10.7759/cureus.73558

**Published:** 2024-11-12

**Authors:** Daria Szczepanek, Ambika Shivarajpur, Eric Boccio

**Affiliations:** 1 Pharmacology, Memorial Healthcare System, Hollywood, USA; 2 Emergency Medicine, Memorial Healthcare System, Hollywood, USA

**Keywords:** adverse drug effect, case report, dress syndrome, drug reaction with eosinophilia and systemic symptoms, trimethoprim/sulfamethoxazole

## Abstract

Drug reaction with eosinophilia and systemic symptoms (DRESS syndrome) is a rare and potentially life-threatening condition. Symptoms typically manifest two to eight weeks after exposure to an offending agent, such as anticonvulsants and antibiotics. Clinical features include fever, morbilliform rash, eosinophilia, lymphadenopathy, and, in severe cases, multiorgan dysfunction, including interstitial nephritis, hepatitis, and pneumonitis.

This case report discusses a 55-year-old female who developed DRESS syndrome while being treated for a urinary tract infection with trimethoprim/sulfamethoxazole (TMP/SMX). Presenting with fever, dysuria, flank pain, and a widespread non-pruritic maculopapular rash, her lab results were remarkable for eosinophilia, while the urinalysis revealed mild hematuria. Computed tomography imaging ruled out nephrolithiasis and acute pyelonephritis, leading to the diagnosis of DRESS syndrome. Management focused on discontinuing TMP/SMX and initiating systemic glucocorticoids. The patient responded well to treatment and was discharged on hospital day two with prescriptions for topical and oral steroids, famotidine, and diphenhydramine. The patient was provided with follow-up instructions and return precautions.

Drug reaction with eosinophilia and systemic symptoms poses unique diagnostic challenges due to its similarity to other cutaneous reactions and the delay between drug exposure and symptom onset. Early recognition and intervention are vital for preventing severe complications. Given its potential for multiorgan dysfunction and poor patient outcomes, healthcare providers must be vigilant when evaluating patients presenting with fever and rash. Comprehensive history taking and accurate reconciliation of active and recent medications are necessary to make the diagnosis. Immediate discontinuation of the offending agent is essential, while supportive care and topical or systemic glucocorticoids remain the treatment standards.

## Introduction

Drug reaction with eosinophilia and systemic symptoms, or DRESS syndrome, is a rare, life-threatening dermatologic drug reaction that affects close to two per 100,000 patients per year and is associated with a 2.9-10% mortality rate [1-2]. Unfortunately, it is often misdiagnosed or mistreated due to its similar presentation to other severe cutaneous drug reactions and its prolonged latency period of two to eight weeks following drug exposure. Drug reaction with eosinophilia and systemic symptoms is a T-cell-mediated drug hypersensitivity reaction characterized by the presence of a rash, fever, eosinophilia, lymphadenopathy, and often systemic multiorgan dysfunction [3]. Cutaneous manifestations typically consist of a morbilliform eruption, maculopapular rash, facial edema, pustules, or purpura, while the involvement of oral and occipital mucosal membranes is rare [4]. We present the case of DRESS syndrome in an adult female secondary to trimethoprim/sulfamethoxazole (TMP/SMX).

## Case presentation

A 55-year-old female with a past medical history inclusive of hypothyroidism and past surgical history inclusive of Cesarean section was recently diagnosed with a urinary tract infection 14 days earlier, having completed a seven-day course of ciprofloxacin and currently on day nine of ten of TMP/SMX presented to the emergency department for fever, dysuria, left flank pain, rash, and facial swelling. The patient reported that she awoke earlier that morning with a non-pruritic rash covering her entire body with associated facial swelling and subjective fever. The patient reported left flank pain radiating to the left lower quadrant and groin. The pain was described as sharp in quality, mild in severity, and constant in duration. The patient denied swelling of the lips and tongue, shortness of breath, cough, and hematuria. The patient reported being post-menopausal and denied any known history of asthma, atopic dermatitis, nephrolithiasis, or known allergies. The patient identified as a never-smoker and refrained from illicit substance and alcohol use. In addition to 800 milligrams (mg) sulfamethoxazole/160 mg trimethoprim per os (PO) twice daily (BID), the patient reported taking 88 micrograms levothyroxine PO once daily (Qd).

**Figure 1:**
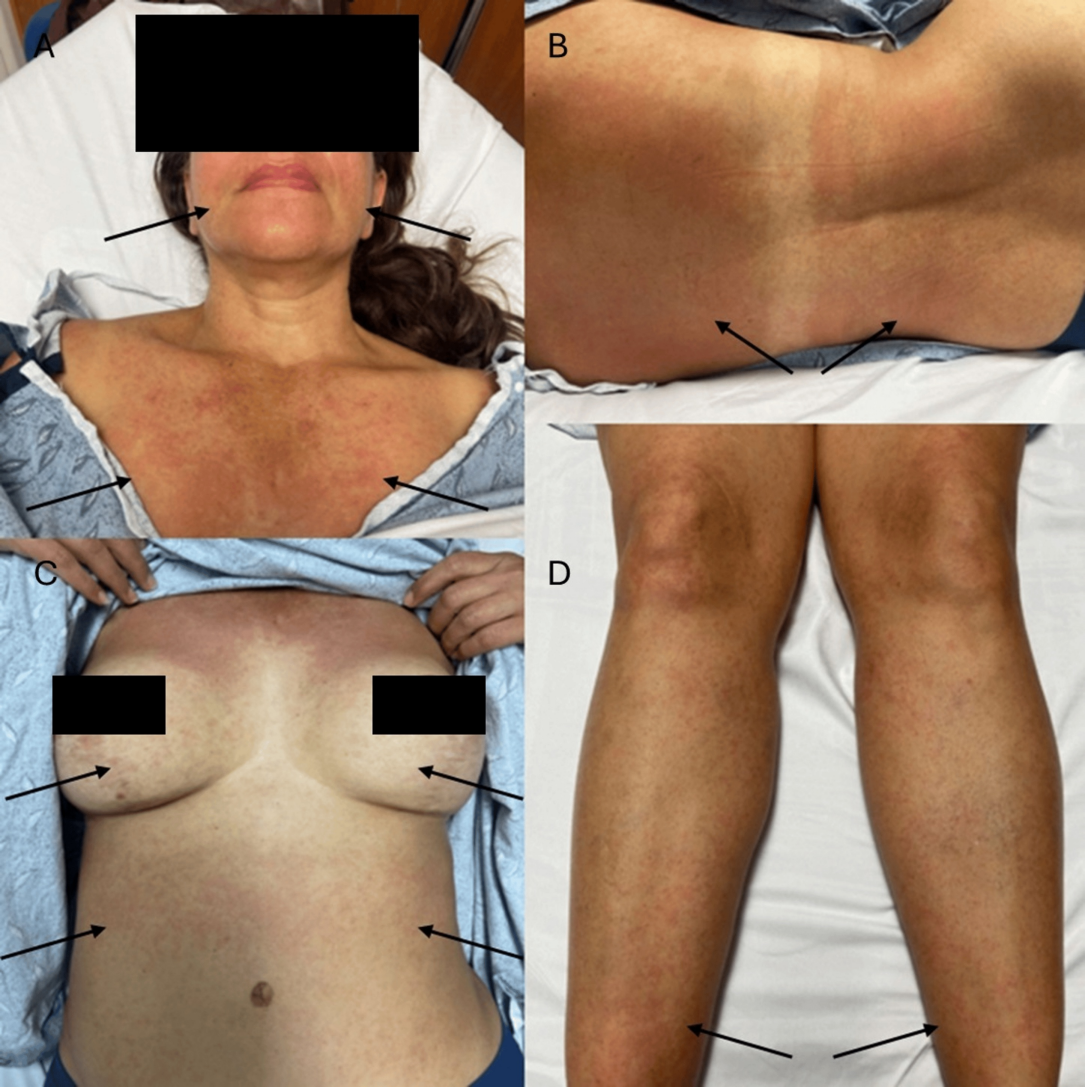
Cutaneous manifestations of DRESS syndrome Physical examination revealed a maculopapular rash on the patient's (A) face and chest, (B) upper and lower back, (C) breasts and abdomen, and (D) bilateral lower extremities (arrows). DRESS syndrome: Drug reaction with eosinophilia and systemic symptoms

Initial vital signs revealed a blood pressure of 118/70 millimeters of mercury, heart rate of 99 beats per minute, respiratory rate of 20 breaths per minute, and temperature of 38.1 degrees Celsius, oral. On arrival, the patient was protecting her airway, spontaneously breathing without signs of difficulty, and was awake, alert, and oriented to self, place, and time. The patient was in no acute distress and appeared nontoxic. Physical examination was remarkable for abdominal tenderness to deep palpation in the left lower quadrant and left costovertebral angle tenderness. There was no rebound tenderness or signs of abdominal distention. A morbilliform rash was present on the patient's face, trunk, abdomen, back, and bilateral lower extremities (Figure 1). The mucous membranes were spared and appeared moist. The anterior cervical lymph nodes were prominent. Auscultation of heart sounds revealed a fast rate and regular rhythm and was free of rubs, gallops, and murmurs. Breath sounds were clear in all lung fields. The remainder of the physical examination was unremarkable.

Given the patient's initial vital signs, history of present illness, physical examination, and suspected urinary source of infection, an institutional sepsis alert was initiated. Corresponding labs were collected, including a complete blood count with differential, basic metabolic panel, urinalysis, urine culture, two sets of blood cultures, and lactic acid. The complete blood count revealed a normal hemoglobin of 11.6 grams per deciliter (g/dL) (11.4-15.4 g/dL) and white blood cell count of 4.0 1000/microliter (µL) (3.5-10 1000/µL). The differential revealed a relative eosinophilia: 43.1% (40-60%) neutrophils, 0.3% (0.0-0.6%) immature granulocytes, 41.3% (18.2-47.4%) lymphocytes, 9.5% (4.3-11%) monocytes, 5.3% (0.0-3.0%) eosinophils, and 0.5% (0.0-0.7%) basophils. Kidney function was within normal limits (blood urea nitrogen 16 mg/dL (7-17 mg/dL), creatinine 0.83 mg/dL (0.51-0.95 mg/dL), glomerular filtration rate (GFR) 83 milliliters/minute/1.73 m^2^ (mL/min/1.73 m^2^) (51-95 mL/min/1.73 m^2^)), and there were no significant electrolyte abnormalities (sodium 137 millimoles/Liter (mmol/L) (137-145 mmol/L), potassium 4.1 mmol/L (3.5-5.1 mmol/L), chloride 106 mmol/L (98-107 mmol/L), calcium 8.8 mg/dL (8.4-10.2 mg/dL)). Lactic acid was within normal limits (0.8 mmol/L (0.7-2.0 mmol/L)). Urinalysis revealed hematuria with 4-5 red blood cells per high power field (cells/HPF) (0-3 cells/HPF). The white blood cell count was within normal limits (0-1 cells/HPF (0-3 cells/HPF)), and no squamous epithelial cells, casts, bacteria, yeast, or crystals were seen. The patient's lab results are summarized (Table 1).

**Table 1:** Summary of patient's laboratory findings Abbreviations: urinalysis (UA), red blood cell (RBC), white blood cell (WBC), grams/deciliter (g/dL), microliter (µL), milligrams/deciliter (mg/dL), milliliter/minute/1.73 meters squared (mL/min/1.73 m^2^), millimole/Liter (mmol/L), high power field (HPF)

Parameter	Patient Value	Reference Range
Hemoglobin	11.6 g/dL	11.4-15.4 g/dL
White blood cell	4.0 1000/µL	3.5-10 1000/µL
Neutrophil (%)	43.1%	40-60%
Immature granulocytes (%)	0.3%	0-0.6%
Lymphocytes (%)	41.3%	18.2-47.4%
Monocytes (%)	9.5%	4.3-11%
Eosinophils (%)	5.3%	0.0-3.0%
Basophils (%)	0.5%	0.0-0.7%
Blood urea nitrogen	16 mg/dL	7-17 mg/dL
Creatinine	0.83 mg/dL	0.51-0.95 mg/dL
Glomerular filtration rate	83 mL/min/1.73 m^2^	51-95 mL/min/1.73 m^2^
Sodium	137 mmol/L	137-145 mmol/L
Potassium	4.1 mmol/L	3.5-5.1 mmol/L
Chloride	106 mmol/L	98-107 mmol/L
Calcium	8.8 mg/dL	8.4-10.2 mg/dL
Lactic acid	0.8 mmol/L	0.7-2.0 mmol/L
UA, RBC	4-5 cells/HPF	0-3 cells/HPF
UA, WBC	0-1 cells/HPF	0-3 cells/HPF

Given the presence of fever, left flank pain, left costovertebral angle tenderness, and hematuria on urinalysis, a CT scan of the abdomen and pelvis was obtained and revealed no evidence of acute pyelonephritis, perinephric abscess, hydronephrosis, or ureteral obstruction bilaterally. Incidentally, mildly prominent stool scattered throughout the colon and several loops of distal small bowel suggestive of constipation were noted.

The patient was given 1000 mg acetaminophen PO, 2 gm ceftriaxone intravenously (IV), 125 mg methylprednisolone sodium succinate IV, diphenhydramine 25 mg IV, famotidine 20 mg IV, and a 30 mL per kilogram bolus (2,200 mL total) of sodium chloride IV. Given the fever, diffuse non-pruritic morbilliform rash which spared the mucous membranes, cervical lymphadenopathy, and eosinophilia, the diagnosis of DRESS syndrome secondary to TMP/SMX was made, and the patient was admitted to the hospitalist service. While admitted, TMP/SMX was discontinued, and 40 mg methylprednisolone IV every 6 hours and 20 mg famotidine PO BID were continued. Infectious disease was consulted, and the suspicion of DRESS syndrome was reaffirmed. The patient was monitored for 24 hours, repeated vital signs and laboratory test results remained unchanged, and the patient was discharged with prescriptions for 1% hydrocortisone topical cream, methylprednisolone taper, 20 mg famotidine PO BID, and 25 mg diphenhydramine PO every 8 hours as needed for two weeks duration. The patient was also prescribed an epinephrine 0.3 mg/0.3 mL autoinjector pen kit and provided with an ambulatory dermatology referral. A review of the patient's medical record revealed no return visits to a clinical care setting within 30 days of the hospital discharge date.

## Discussion

Drug reaction with eosinophilia and systemic symptoms has an estimated prevalence of 2.18 per 100,000 cases [1-2]. Drug reaction with eosinophilia and systemic symptoms syndrome typically affects adults 20 to 50 years of age, with a slight predominance in females and the black population [3-4]. Risk varies across medication types, with antibiotics and anticonvulsants representing the majority of primary drug culprits (23-74% and 20-35%, respectively) [1,5]. Common antibiotics that have been associated with DRESS syndrome include vancomycin (39%), penicillin (13%), cephalosporins (7%), tetracyclines (4%), sulfonamides (3%), and carbapenems (3%) [1,4]. A causative drug cannot be identified in approximately 10-20% of cases [5].

Drug reaction with eosinophilia and systemic symptoms typically presents two to eight weeks after drug exposure, with a reported median time interval after drug intake of 22 days [5-7]. The acute phase of DRESS syndrome is characterized by a flu-like prodrome, the development of a skin rash, and facial edema [8]. During this period, activated T lymphocytes produce cytokines such as tumor necrosis factor-alpha, interleukin-6, and interferon-gamma, which are responsible for the pro-inflammatory state and organ dysfunction associated with severe DRESS syndrome [9]. Reactivation of a virus, commonly Human herpesvirus-6, mumps, Epstein-Barr virus, and cytomegalovirus, has been shown to exacerbate DRESS syndrome [6-7]. Genetic variations in specific human leukocyte antigens are associated with increased susceptibility and most commonly affect those of Asian and European descent [3,10].

High-risk medications like TMP/SMX trigger the release of granulysin, an extremely potent cytolytic cytokine from cytotoxic T-cells and natural killer cells. The destruction of cells in the epidermal and dermal skin layers results in the cutaneous manifestations commonly seen [11]. N-acetyl-sulfamethoxazole, an active metabolite of SMX, is postulated to activate the immune cells responsible for DRESS syndrome secondary to TMP/SMX [12]. In some cases, multiple systemic antibiotics pose a challenge in identifying the causative agent. The inability to differentiate between an immediate hypersensitivity reaction mediated by immunoglobulin E and a delayed T-cell-mediated reaction may result in a delayed or missed diagnosis and inappropriate or unnecessary therapeutic management [11].

Clinically, the acute phase of DRESS syndrome, which occurs zero to 11 days following drug initiation, is associated with fever accompanied by a maculopapular rash involving the face, trunk, and lower extremities [6]. Periorbital and facial edema with pustules may be present [7]. Typically, mucosal surfaces, as well as the palms and soles, are spared, distinguishing DRESS syndrome from Stevens-Johnson Syndrome (SJS) and Toxic Epidermal Necrolysis Syndrome (TENS) [7]. Severe systemic features of DRESS syndrome include lymphadenopathy, hematological abnormalities such as eosinophilia, thrombocytopenia, and pancytopenia, and multiorgan dyscrasias including hepatitis, nephritis, pneumonitis, and myocarditis [6,8]. Unlike SJS and TENS, DRESS syndrome more commonly affects the liver and kidneys, with abnormal liver function tests mimicking viral hepatitis and kidney injury attributed to acute interstitial nephritis [4,13].

The mainstay of treatment is discontinuing the offending agent and supportive care [6]. Standard treatment for symptom management includes systemic glucocorticoids [8]. Steroid-sparing agents, including cyclosporine, mycophenolate mofetil, and IV immunoglobulin G, have also proved successful [13]. An allergy workup may be beneficial in determining the culprit drug and potential sensitivities to medication cross-reactions [14]. Given the cross-reactivity seen with sulfonamides, patients who experience DRESS syndrome secondary to TMP/SMX should avoid dapsone and sulfasalazine, and empiric treatment with penicillins and non-steroidal anti-inflammatory drugs should be exercised with caution [14-15]. In patients with mild DRESS syndrome, topical steroid treatment and close follow-up are recommended. In patients with severe DRESS syndrome, which includes systemic multiorgan dysfunction such as liver, lung, or acute kidney injury, systemic glucocorticoid treatment and admission to intensive care environments may be warranted. If systemic glucocorticoid treatment is contraindicated, cyclosporine is the recommended treatment [14]. Other aspects of supportive care for DRESS syndrome include close monitoring, electrolyte and fluid repletion, anticoagulation prophylaxis as needed, gastric protection, fever and pain control, and topical skin care treatments [14]. On average, the recovery period is approximately six to nine weeks; however, persistent relapses have been reported in more than 20% of cases [16]. Risk factors for DRESS syndrome-specific mortality include an absolute eosinophil count above 6000 1000/µL, thrombocytopenia, pancytopenia, leukocytosis, coagulopathy, and a past medical history of chronic kidney disease [17].

## Conclusions

Drug reaction with eosinophilia and systemic symptoms is a rare but potentially life-threatening drug reaction typically induced by antibiotics such as sulfonamides, anticonvulsants, and allopurinol. Clinically, it is characterized by fever, an extensive and often exfoliative skin rash, lymphadenopathy, and eosinophilia, and, in severe cases, it is often accompanied by organ dysfunction, particularly hepatitis, renal impairment, and pneumonitis. The typical onset occurs two to eight weeks following exposure to an offending drug, making timely identification a diagnostic challenge. An increased awareness, comprehensive history taking, and an accurate reconciliation of active medications are essential in diagnosing. Early intervention, including drug withdrawal, supportive care, and topical or systemic glucocorticoid treatment, is crucial to mitigate severe complications and improve patient outcomes.

## References

[1] Wolfson AR, Zhou L, Li Y, Phadke NA, Chow OA, Blumenthal KG (2019). Drug reaction with eosinophilia and systemic symptoms (DRESS) syndrome identified in the electronic health record allergy module. J Allergy Clin Immunol Pract.

[2] Hiransuthikul A, Rattananupong T, Klaewsongkram J, Rerknimitr P, Pongprutthipan M, Ruxrungtham K (2016). Drug-induced hypersensitivity syndrome/drug reaction with eosinophilia and systemic symptoms (DIHS/DRESS): 11 years retrospective study in Thailand. Allergol Int.

[3] Cheng CY, Su SC, Chen CH, Chen WL, Deng ST, Chung WH (2014). HLA associations and clinical implications in T-cell mediated drug hypersensitivity reactions: An updated review. J Immunol Res.

[4] Jeung YJ, Lee JY, Oh MJ, Choi DC, Lee BJ (2010). Comparison of the causes and clinical features of drug rash with eosinophilia and systemic symptoms and stevens-johnson syndrome. Allergy Asthma Immunol Res.

[5] Kardaun SH, Sekula P, Valeyrie-Allanore L (2013). Drug reaction with eosinophilia and systemic symptoms (DRESS): An original multisystem adverse drug reaction. Results from the prospective RegiSCAR study. Br J Dermatol.

[6] Cardones AR (2020). Drug reaction with eosinophilia and systemic symptoms (DRESS) syndrome. Clin Dermatol.

[7] Shiohara T, Mizukawa Y (2019). Drug-induced hypersensitivity syndrome (DiHS)/drug reaction with eosinophilia and systemic symptoms (DRESS): An update in 2019. Allergol Int.

[8] Hama N, Abe R, Gibson A, Phillips EJ (2022). Drug-induced hypersensitivity syndrome (DIHS)/drug reaction with eosinophilia and systemic symptoms (dress): Clinical features and pathogenesis. J Allergy Clin Immunol Pract.

[9] Criado PR, Criado RF, Avancini JM, Santi CG (2012). Drug reaction with eosinophilia and systemic symptoms (dress) / drug-induced hypersensitivity syndrome (DIHS): A review of current concepts. An Bras Dermatol.

[10] Hung SI, Chung WH, Jee SH (2006). Genetic susceptibility to carbamazepine-induced cutaneous adverse drug reactions. Pharmacogenet Genomics.

[11] Lin YF, Yang CH, Sindy H (2014). Severe cutaneous adverse reactions related to systemic antibiotics. Clin Infect Dis.

[12] Sharifzadeh S, Mohammadpour AH, Tavanaee A, Elyasi S (2021). Antibacterial antibiotic-induced drug reaction with eosinophilia and systemic symptoms (DRESS) syndrome: A literature review. Eur J Clin Pharmacol.

[13] Mąsior MN, Rostkowska OM, Furmańczyk-Zawiska A, Wieczorek-Godlewska R, Wyzgał M, Durlik M (2024). Dress syndrome: Renal involvement in two cases - a comprehensive analysis and literature review of improved diagnosis and treatment. Am J Case Rep.

[14] Cabañas R, Ramírez E, Sendagorta E (2020). Spanish guidelines for diagnosis, management, treatment, and prevention of DRESS syndrome. J Investig Allergol Clin Immunol.

[15] Shiohara T, Inaoka M, Kano Y (2006). Drug-induced hypersensitivity syndrome (DIHS): A reaction induced by a complex interplay among herpesviruses and antiviral and antidrug immune responses. Allergol Int.

[16] Calle AM, Aguirre N, Ardila JC, Cardona Villa R (2023). DRESS syndrome: A literature review and treatment algorithm. World Allergy Organ J.

[17] Husain Z, Reddy BY, Schwartz RA (2013). DRESS syndrome: Part I. Clinical perspectives. J Am Acad Dermatol.

